# The relationship between health promoting resources and work participation in a sample reporting musculoskeletal pain from the Nord-Trøndelag Health Study, HUNT 3, Norway

**DOI:** 10.1186/1471-2474-14-100

**Published:** 2013-03-19

**Authors:** Heidi Sivertsen, Monica Lillefjell, Geir Arild Espnes

**Affiliations:** 1 Department of Social Work and Health Science, Norwegian University of Science and Technology, NTNU, Trondheim, Norway; 2 Research Centre of Health Promotion and Resources, HIST/NTNU, Trondheim, Norway; 3 Department of Occupational Therapy, Sør Trøndelag University College, HIST, Trondheim, Norway

**Keywords:** Musculoskeletal pain (MSP), Health promotion, Resources, Salutogenic, Work- participation

## Abstract

**Background:**

Musculoskeletal pain (MSP) is one of the most frequent causes of sick leave from work, and is a common and potentially disabling condition. This study is based on the salutogenic perspective and investigates the relationship between personal, social, and functional health resources and work participation in a population reporting MSP.

**Method:**

Analysis was performed on cross sectional data from the Nord-Trøndelag Health Study, HUNT 3, in Norway. The sample of n= 6702 was extracted from HUNT 3, including a total of N= 50807 participants. Self-reported health (SRH) and, personal, social, and functional resources were assessed by a questionnaire. Reported sick leave was collected by interview at the point of time when the data were collected, from October 2006 until June 2008.

**Results:**

Logistic regression analysis demonstrated statistically significant differences between the work group and sick leave group in self-rated health, work support, work control, work load, and feeling strong, and the model predicted 68% of the cases correctly. Females had a lower statistically significant probability (*B*= −.53) to be in the work group then men when suffering from MSP, with odds of 41%.

**Conclusion:**

There was a statistically significant relationship between health promoting resources such as SRH, feeling strong, absence of neuroticism, work load, work control, and work participation in MSP population.

## Background

Suffering from musculoskeletal pain (MSP) can be seen as being on the spectrum from health to disease [1]. MSP and illnesses related to MSP are the most frequent causes of sickness and disability from work, and MSP is a common and potentially disabling condition in western societies [2-4]. The World Health Organization defines health as “personal and social resources as much as physical capacity that realizes experiences in life as meaningful and provides creative and productive members of society” [5]. Therefore, a full comprehension of musculoskeletal health calls for an understanding of what contributes to a better health, and what contributes to the development of disease.

Traditionally, health concepts for absenteeism, work disability, and returning to work have been built on the pathological model [6]. In the same way, public health and health promotion have developed theories on what minimizes pathology; how to identify factors that combat disease and improve health [7,8]. As a result, most area knowledge focuses on MSP and risk factors predicting pain and illnesses [9-11], rather than on factors predicting health improvement [8,12,13], especially for working individuals with MSP [14,15]. The consequence is that individuals seek help for outward relief (e.g. medication), instead of taking part in the active process of making healthier choices. In order to identify and reduce the impact of MSP, there is a need for more knowledge regarding factors associated with a good health outcome, and an investigation of combinations of personal, social, and functional resources that have a relation to work participation [8,12].

This study is based on the concept of a salutogenesis, defined by Aaron Antonovsky as the process towards the health end of a health ease/ disease continuum [12]. In salutogenic theory, the first key feature is how individuals meet life challenges with a degree of comprehensibility, manageability and meaningfulness according to their available resources [12]. The other key feature of the salutogenic theory is generalized resistance resources (GRRs). These are the prerequisites for the development of Sense of Coherence (SOC), and are found in personal skills and in an individual’s immediate or distant environments [12]. The level of coping capacity depends on the GRRs, and a resource is a personal or environmental factor that promotes health [8,12].

Antonovsky (1987) has proposed eight types of GRRs. These are physical, biochemical, material, cognitive, and emotional, and they are also values/attitudes, interpersonal relations and macro socio-cultural resources that work together to deal with the tension of stressors. Variables such as self-esteem, social support, high social class, and cultural stability are examples of GRRs [12]. How available resources are used in a manner promoting health is essential for a salutogenic result [8]. This study aim is to investigate the relationship between health promotion resources and work participation in a population reporting MSP.

## Methods

The data used, were provided from the Health Survey of Nord-Trøndelag (HUNT), Norway. HUNT is considered one of the largest health surveys in the world, and is well suited to epidemiological research because of the stabile and homogenous population [16]. The third health survey in Nord-Trøndelag (HUNT 3) was conducted from October 2006 to June 2008. Approximately 105,000 inhabitants were invited by a self-administered questionnaire, sent through the mail. The participation rate was 50 807 (49%). The HUNT study and this study were approved by the Regional Committee for Medical and Health Research Ethics (REK), Norway.

### Participants and settings

For this study, participants included women and men (20–69 years) in the working age population reporting MSP over the last year (n=6702). The inclusion criteria were that participants have had pain or stiffness in muscles or joints that lasted at least 3 consecutive months, had a job, and answered “moderate”, “strong” or “very strong” on the question: “How strong has your physical pain been during the last 4 weeks?”. All participants who answered “Yes” or “No” to the question “Have you been on sick leave in the past 12 months”, were included to represent outcome variable as the work group and the sick leave group (with/without certified sick leave from doctor) in the study. The total data material consisted of N = 50 807 with a prevalence of musculoskeletal pain (MSP) or stiffness in muscles or joints that had lasted at least 3 consecutive months of 39.5% (n=20 051). The final sample included only those participants who reported “moderate” to “very strong” pain, n=6702, and those who had answered the question about sick leave. Missing were 1.6%.

In spite of the gross measure of pain, both the work group and sick leave group showed homogeneity according to reporting moderate, strong, or very strong pain. The work group reported a 78.1% occurrence rate of moderate pain, 20.1% of strong pain, and 1.8% of very strong pain, respectively 78.8%, 19.3% and 1.9% in the sick leave group. The nature of MSP, and the fact that the work group and sick leave group were homogeneous, would counteract the gross measure in sick leave.

### Personal resources

The independent variables were selected based on the salutogenic theory [8], and supported empirically from the resilience research within the dimensions of personal, social, and functional resources [17]. Personal resources were measured by a 12 items short form of the Eysenck Personality Questionnaire (EPQ) scale [18]. The items were as follows: 1)“Are you a life of the party type of person;” 2)“Are you mostly quiet and reserved when you are around other people;” 3)“Describe yourself as you normally are;” 4)“Do you like meeting new people;” 5)“Do you like to have a lot of life and excitement around you;” 6)“Are you a relatively lively person;” 7)“Do you usually take the first step to make new friends;” 8)“Are you often worried;” 8)“Are your feelings easily hurt;” 9)“Do you often feel that you lose interest;”10)“Do you have nervous problems;” 11)“Do you often feel tired and indifferent/unmotivated without reason” and 12)“Do you worry that terrible things might happen.” The response options were “No” and “Yes.”In short, six items represented extroversion, known as positive affects, and six items represented neuroticism, known as negative emotions. The meaning variable was measured by a single item: “When something bad happens in my life, I think that is happen for a purpose,” with response options “No,” “Yes,” and “Don’t know.”

### Social resources

Social resources were measured by a single question of social support: “Do you have friends that can help you when you need them,” with the response options “No,” and “Yes.” Social cohesion was measured by, “Do you have friends that you can speak to confidentially,” with the response options “No” and “Yes.” Social activities were measured by six items, namely, “How many times in the last 6 months have you participated in an association or club meeting/activity – in music, singing or theatre – in parish work – in outdoor activities – in dance and in sports or worked out”. Each item had four response options from “more than “1×/week” to “never.”

### Functional resources

Functional resources were measured as physical exercise and self-rated health (SRH). Present health status was measured by a single question health indicator, which was “How is your health now,” with four response options from “poor” to “very good?” Physical exercise was measured by one question: “How often do you exercise?” with the response options on a scale from 1–5, from “never” to “nearly every day.” Feeling strong was measured by, “Do you feel for the most part, strong and fit or tired or worn out?” The response options were on a scale from 1–7, from “very strong and fit” to “very tired and worn out.” The question was reversed so high scores indicated strength and a feeling of being fit.

### Work resources

Work characteristics were measured with 12 items containing different personal, social, and functional resources and were reduced by factor analysis [19]. The items were as follows:

(1) “There is a good collegiality at work;” (2) My co-workers are there for me (support me);” (3) I get along well with my co-workers;” (4) “Does your job require you to work very fast;” (5) “Does your job require you to work very hard;” (6) “Does your job require too great a work effort;” (7) “Do you have the possibility to decide for yourself how to carry out your work;” (8) “Do you have the possibility to decide for yourself what should be done in your work;” (9) “Is your work so physically demanding that you are often physically worn out after a long day’s wor7k;” (10) “Are you bullied/harassed at work;” and (11) “Does your job require creativity.” The last item 12) was a single question only for the age range of 20–29 years “All things considered, how much do you enjoy your work.” For all questions the response options were divided into four point likert scale indicating agreement or disagreement.

The data used in this study was collected by a self administered mail questionnaire, and reported sick leave was the only question in this study administered as an interview during the clinical examinations administered by the HUNT research centre, and registered in the HUNT data bank.

### Statistical analysis

The statistical analysis was first used testing for assumptions of normally distributed data to meet criteria for parametric tests. Factor and reliability analysis was used to determine the suitability of constructing scales, and composite scores of means were made when appropriate. Gender was included in the analysis.

Factor analysis for the Eysenck Personality Questionnaire (EPQ) obtained a factor solution through (direct oblimin rotation) a structure of 6 items in an extroversion (EPQ- E) scale, and 6 items in a neuroticism (EPQ-N) scale. The KMO and Bartlett’s test was .809. Both EPQ-E and EPQ-N achieved alpha coefficients well in excess of .73 and .74, respectively.

Work characteristics (12 items) were analyzed by factor analysis and obtained three component loadings (with direct oblimin rotation), which explained 62% of the variance; work support explained 26.5% , work load 19.5% and work control 16%. The Cronbach’s alpha were .869, .817, and .747, respectively. The first component work support loaded on item 1), 2), 3), and 10) explained 26.5% of the variance of work, and the second component work load loaded on question 4), 5), 6), and 9) explained 19% of the variance of work. The last work control component loaded on question 7) and 8) explained 16% of the variance of work.

The variable work support was reversed so that the high score was “strongly agree,” and the variable work control was reversed so that a high score was “yes, often.” The items 10), 11), and 9) were excluded in factor analysis through reliability analysis because Cronbach’s alpha increased from .489 to .869, from .697 to .817 and from .721 to .747 respectively, which indicate that these items are measuring something else. The item 12) had too few cases to be included in the analysis. The work support variable and work control variable were reversed so that high scores indicated greater support and greater level of work control, to ease the following analysis. All three variables were used as mean scores of multiple items. In summary, the work load component included working “hard” and “fast,” work control included deciding “what” and “how” work should be done, and work support included “well-being” and “support.”

Further, ordinal variables with more than four levels were treated as continuous, due to the large sample size. Bivariate analysis was obtained with different types of Pearson’s correlation coefficient relevant to the present level of measurement. Mann–Whitney tests were used for group comparisons (work and sick leave) because of unequal group size and violation of the homogeneity of variance assumption.

Crosstabs and chi-square statistics (Phi and Pearson) were used to analyze categorical variables relationships and differences between groups. We included only covariates that were significantly associated with SRH, and the statistical significant variables from bivariate analysis in the multivariable logistic regression model. Finally, logistic regression was used to formulate a model about health promoting resources that might determine whether a person with pain is working or being sick listed. For all analysis, a significance level of *p=*.01 was selected to evaluate the significance of the results. Data was analyzed using SPSS version 19.

## Results

This study aimed to investigate the relationship between health promotion resources and work participation in a population reporting musculoskeletal pain. The work group represented 32% (n=2161) and was fairly equally distributed with 50.6% female (n=1094) and 49.4% men (n=1067), and with a mean age of 51.29 (*SD*= 9.68). The sick leave group represented 67% (n=4511) and consisted of 65.1% (n=2935) female and 34.9% (n=1576) men, with a mean age of 49.9 (*SD*= 9.95).

The distribution of MSP showed an equal pattern between the groups with shoulder pain (60%) in the working group and in the sick leave group (64%); neck pain was reported by 56% versus 60%; pain in lumbar regions was reported by 50% versus 54%; and pain in hips was reported by 35% versus 41%. The K-S test for normality was (*D*(2129) =.082, *p*<.001) for the work group and (*D*(4427) =.078, *p*<.001) for the sick leave group, which indicated deviation from normality. Descriptive results were presented in Table 1 with grouping the variable “Have you been on sick leave in the past 12 months?” (n=6672).

**Table 1:** Descriptive statistics over study variables, total sample n=6702

**Variables**	**n**	***M***	***SD***	**Missing n (%)**
*Work group*				
Physical exercise (range 1–5)	2154	3.40	1.10	7 (0.3)
EPQ extroversion (range 0–6)	2062	3.73	1.83	99 (4.6)
EPQ neuroticism (range 0–6)	2087	1.76	1.72	74 (3.4)
Feeling strong (range 1–7)	2142	4.63	1.12	19 (0,9)
Social activities (range 1–5)	2148	2.05	.75	13 (0,6)
Work support (range 1–4)	1972	3.38	.53	189 (8.7)
Work load (range 1–4)	2029	2.08	.65	132 (6.1)
Work control (range 1–4)	2029	3.27	.75	132 (6.1)
*Sick leave group*				
Physical exercise (range 1–5)	4498	3.45	1.06	13 (0,3)
EPQ extroversion (range 0–6)	4300	3.83	1.78	211 (4.7)
EPQ neuroticism (range 0–6)	4350	2.17	1.85	161 (3.6)
Feeling strong (range 1–7)	4465	4.25	1.15	46 (1.0)
Social activities (range 1–5)	4485	2.09	.76	26 (0,6)
Work support (range 1–4)	4209	3.26	.56	302 (6.7)
Work load (range 1–4)	4258	2.01	.63	253 (5.6)
Work control (range 1–4)	4243	3.06	.78	268 (5.9)

### Work group and sick leave group comparisons

A Mann- Whitney *U* test was conducted to evaluate nine resources (physical exercise, EPQ-E and N, feeling strong, social activities, work support, work load and work control) if there were any differences between the work group and the sick leave group. A significant difference was found in levels of SRH between the work group and the sick leave group, *p* <.001. No differences were found between the two groups in levels of physical exercise, social activities or levels of EPQ-E. EPQ-N levels were significantly higher in the sick leave group than in the work group, *p* = <.001. Further, the work group, reported significantly higher levels of feeling strong than the sick leave group, *p* = <.001. The work group reported higher levels of work support than the sick leave group, *p* = <.001, and the work group reported higher levels of work load than the sick leave group, *p* = <.001. Moreover, the work group reported higher levels of work control than the sick leave group, *p* = <.001. This effect, however, was below the .03 criterion for a medium effect and could be considered a small effect [20]. The total results were presented in Table 2.

**Table 2:** Mann–Whitney U test results between the groups

	**M work group**	**M sick leave group**	**U**	***p***	***r***
SRH	3688.73	3081.22	3839165.00	.001	-. 17
Physical exercise	3269.32	3353.88	4721185.00	ns	-.02
EPQ-E	3127.04	3207.62	4320999.00	ns	-.02
EPQ-N	2945.66	3350.14	3968762.50	.001	.10
Feeling strength	3728.72	3100.25	3872258.00	.001	-.16
Social activities	3250.99	3348.62	4675090.00	ns	-.02
Work support	3341.21	2973.77	3656664.00	.001	-.10
Work load	3266.74	3085.51	4070700.00	.001	-.05
Work control	3472.06	2976.04	3623673.00	.001	-.13

Variable relationships were assessed thorough chi-square statistics (phi) and showed significant gender difference between the groups, *x*^*2*^(6672) = −.138, *p* = < .001. Only the significant variables that were seen as health promoting resources as SRH, feeling strong, work support, work load and work control were included for further analysis.

### Meaning, friends’ support and cohesion

Three categorical variables (meaning, friends’ support and friends’ cohesion) were assessed with chi-square statistics (Phi for 2 × 2 Tables and Pearson). There was significant gender difference between the two groups, with more men in the work group, *x*^*2*^(6672) = −.138, *p* = < .001. No significant difference was found between the work and sick leave groups in the meaning variable, *x*^*2*^(3) = 2.935, *ns*.

The work group reported slightly more support at work (95.4%) than the sick leave group (95.1%) (*x*^*2*^(1) = −.008, *ns)*. However, participants in the sick leave group reported significantly more friends’ cohesion (91%) than participants in the work group (89%)(*x*^*2*^(1) = −.29, *p* <.005).

**Figure 1:**
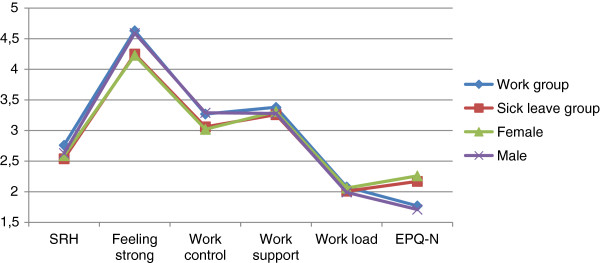
Work and sick leave group, female and men composite scores of means.

For further analysis, the variables EPQ-E, meaning, and friends support were not included because of non significance differences between groups. Friends’ cohesion was not included because of the preliminary findings and a small difference. For constructing a model of prediction for the work group, only variables with statistically significant group and gender differences in means were presented in Figure 1.

### Model of work with logistic regression analysis

According to the aim of the study and preliminary findings, a logistic regression analysis was conducted to predict belonging to the work group using five variables (SRH, feeling strong, work support, work load, and work control) as independent associations. Age and gender were included. A test of the full model (forward LG method) against a constant only model was statistically significant, indicating that the independent associations are a set, reliably distinguished between the work group and sick leave group (chi-square = 408.264, *p*=000 with (*df* = 7)). Nagelkerke’s (*R*^*2*^*of .092)* indicated a weak relationship between prediction and grouping. Prediction success overall was 68%, which was more than by chance. The model fit is acceptable (*x*^*2*^(8) =10.973, *p*=.203). The Wald criterion (with acceptable *S.E)* demonstrated that only SRH made a significant contribution to prediction (*p=*.0001) of the work group. Collinarity statistics were satisfied, and Leverage values were satisfyingly low.

Due to the subtle differences between each step, the main outcomes were reported in the last step. *EXP(B)* indicated that when SRH raised with one unit, the odds to be in the work group increased by 66%. *EXP(B)* for work support, work control, and work load indicated that when these variables increased by one unit each, the odds to be in the work group increased by 29%, 21%, and 16%, respectively. *EXP(B)* for the feeling of being strong indicated that when it increased by one unit, the odds to be in the work group increased by 12%. According to gender, we see that females had a statistically significant lower probability (*B*= −.53) to be in the work group than men, with an odds of 41%. The total results were presented in Table 3.

**Table 3:** Work group prediction model with logistic regression

**95% CI for exp *b***				
**Included (last step)**	***B *( *SE *)**	**Lower**	**exp *b *(%)**	**Upper**
Constant	4.60**(0,28)	NA	.01	NA
SRH	.51 **(0,05)	1.49	1.66 (66)	1.84
Feeling strong	.12**(0,03)	1.06	1.12 (12)	1.19
Work load	.15* (0,05)	1.06	1.16 (16)	1.27
Work control	.19**(0,04)	1.12	1.21 (21)	1.31
Age	.15**(0,03)	1.09	1.16 (16)	1.24
Work support	.25**(0,06)	1.16	1.29 (29)	1.44
Gender	-.53**(0,06)	0,53	0,59 (41)	0,66

Note: n= 6702. Levels of significance: *p<.001, **p<.0001.

## Discussion

In our study, we found a relation between health promoting resources and work participation, consistent with theoretical assumptions. These resources could strengthen individuals’ health and promote work participation, despite moderate to very strong pain. The main findings were that SRH and gender (men) were the variables which best predicted work participation with MSP. SRH are well known as a reliable health promoting resource [21].

There were differences between participants in the work group and the sick leave group in health promoting resources, such as in feeling strength or in absence of neuroticism. Effective pain management recognizes the importance of functional factors, but also emphasizes the influences that psychological factors (e.g., anxiety, perceived control) and social factors (e.g., family and work environment) can have on the experience of pain [22]. Attitudes and beliefs are relevant in relation to work participation, but it is beyond the scope of this study.

### Personal resources and health promoting effect

The results for personal resources were consistent with empirical findings in resilience research [23]. Resilient individuals tended to be characterized by higher extraversion and lower neuroticism levels. Extroversion predicted effective functioning across a wide array of domains from aging to responses to loss [23]. In this study, both the work and the sick leave groups reported similar levels in extroversion, but differed significantly in levels of neuroticism. This indicated that extroversion personality levels did not outweigh the experience of moderate to strong pain, but there was a statistically significant probability that absence of neuroticism did [24].

Neuroticism is a relative stable personality trait, and individuals who score high on neuroticism are predisposed to experiencing feelings as general emotionality, impulsivity, fear, anger, and psychosomatic concerns [25]. Using a multidisciplinary approach could reveal mechanisms that produce the stable and mutable components between neuroticism and psychopathology [26], and is especially recommended for MSP patients with low back pain sick listed above 12 weeks [9]. The differences between groups in neuroticism were in line with the salutogenic theory, in which Antonovsky (1987) characterized solution processes as cognitive and emotional expectations of life as something comprehensible, manageable, and meaningful. This was quite different than what the characteristics of neuroticism produced. These results also supported earlier empirical results and theories behind neuroticism, which block up for pain relief in individuals who started off with a chronically higher level of arousal than extroverts [27].

One question that remains is however, is it possible that health promoting effect in pain distraction lies in the absence of neuroticism qualities, and not, as assumed, in extroversion qualities? This question is beyond the scope of this study to answer, but our findings point out these issues as relevant to follow up in future studies.

### Social resources and health promoting effects

Work support was a health resource across the two groups and was recently supported by a qualitative study about success factors for staying at work with MSP [6]. Positive emotions through social support are a basic building block obtained through colleagues. The feeling of support promotes flexibility in thinking and problem solving, counteracts the physical effects of negative emotions, and promotes adaptive coping [23,28,29]. Social support was probably the most empirically documented resource for adjustment, resilience, and health. The difference in this study, between female and male was only one fourth of the difference between the work group and sick leave group, which indicated that work support in the work group not could be explained with gender differences, as we tend to do. The difference between female and male was quite small.

### Functional resources and health promoting effect

The SRH was characterized in this study to include many different variables measuring different aspects, and was capable of predicting 68% of the work group. The purpose of self-assessment of health was to reflect health trajectories as personal and social resources [30] and it provided a formal means for the individual’s judgment to influence health. A consistent finding is that very simple summary health ratings hold surprising predictive validity for health [30]. Further, sick leave could have been caused by other health problems or diseases than MSP, since we did not control for other diagnoses.

There was a statistically significant difference between the work group and sick leave group in the feeling of strength, and was just as equally distributed within gender. The work group reported higher levels of strength, than both females and males separately. This difference between groups could not be explained by gender differences. Salutogenic theory focuses on the tension between internal and external resources and demands [12]. Emotions are an important component in coping, and satisfying demands in the working life are a position of tension for the individual, where a feeling of strength could be an important resource.

According to Antonovsky, this tension can only be sorted out in a two-fold way, through problem-solving and regulation of emotions [12]. It is possible that a feeling of strength is a result of the absence of neuroticism. The pain may not completely disappear, but the feeling is more optimistic and proactive in finding ways to manage pain, and can improve working capacity and health.

Work load (working hard and fast) and work control (deciding what work and how it should be done) were predictors of being in the work group. To enable work participation with pain, the contingency to do modifications at work and shape suitable work conditions to decrease work load was important [6]. Antonovsky supported the notion that work played a significant role in for a person’s GRR’s with a work environment, which was predictable, and manageable in that the employee could participate in decision making in regulating the work, which enhanced the coping ability of the employee because work is meaningful [12].

### Limitations and strengths of the study

However, the study has a number of potential limitations that may restrict the generalization of the findings. First and most important, causal inferences cannot be drawn from cross sectional data. Second, because survey data is self-reported, they are subject for recall bias and over- and/or under estimating. Third, the self reported sick leave measure was a very gross measure and it is not possible to differentiate in the survey data between participants who have been out for one day with the flu, or out for several months with MSP but the nature of MSP, with the fact that the work group and sick leave group are homogeneous, would counteract this bias.

Type of occupation was not taken into account, and work related MSP could be a potential bias for the results. This may have affected the results in a way that females with heavy work and work-related MSP, may have been interpreted as females with a lack of health promoting resources in this study.

The models specified and tested in two independent samples supported the research question that there are associations between health promoting resources such as SRH, feeling strong, absence of neuroticism, work load, work control, and work participation in people reporting MSP. The study had a cross sectional design and the results were principally based on correlation analysis and logistic regression analysis, and have evoked a new hypothesis about work participation with MSP.

The strength of the current study is the sample size, with over 50 000 participants who had completed a comprehensive range of assessments, including one established instrument which is EPQ. Self reported health holds surprising predictive validity for health [21], and self reported sick leave measures were found to be good to fair between self-report and official registered data on sick leave [31].

## Conclusions

### Implications for health promotion and future research

There was a statistically significant relationship between health promoting resources such as SRH, feeling strong, absence of neuroticism, work load, work control, and work participation in MSP population. In general, knowledge of resources, predicting good health and work participation, should be used to optimize treatment strategies and health promotion programs for subjects with and without MSP. Although our data indicate associations between health promoting resources and work participation in people reporting MSP, it is important to provide further evidence on the causal relationship between these variables, using longitudinal and prospective studies in which changes in each variable may be controlled for over time with diagnostic criteria and/or and sick leave measures.

## Abbreviations

HUNT: The Nord-Trøndelag Health Study

## Competing interests

The authors declare that they have no competing interests with regard to this work.

## Authors’ contributions

HS conceived the study, carried out the statistical analysis and drafted the manuscript. ML and GAE participated in its design and coordination and helped to draft the manuscript. All authors read and approved the final manuscript.

## Pre-publication history

The pre-publication history for this paper can be accessed here:

http://www.biomedcentral.com/1471-2474/14/100/prepub
